# Calcium Balance in Chronic Kidney Disease

**DOI:** 10.1007/s11914-017-0368-x

**Published:** 2017-05-04

**Authors:** Kathleen M. Hill Gallant, David M. Spiegel

**Affiliations:** 1 Department of Nutrition Science, Purdue University, West Lafayette, IN USA; 2 Clinical Development, Relypsa, Inc., Redwood City, CA USA

**Keywords:** Calcium, Calcium balance, Chronic kidney disease, Renal failure, Chronic kidney disease-mineral bone disorder, Nutrition

## Abstract

**Purpose of Review:**

The kidneys play a critical role in the balance between the internal milieu and external environment. Kidney failure is known to disrupt a number of homeostatic mechanisms that control serum calcium and normal bone metabolism. However, our understanding of calcium balance throughout the stages of chronic kidney disease is limited and the concept of balance itself, especially with a cation as complex as calcium, is often misunderstood. Both negative and positive calcium balance have important implications in patients with chronic kidney disease, where negative balance may increase risk of osteoporosis and fracture and positive balance may increase risk of vascular calcification and cardiovascular events. Here, we examine the state of current knowledge about calcium balance in adults throughout the stages of chronic kidney disease and discuss recommendations for clinical strategies to maintain balance as well as future research needs in this area.

**Recent Findings:**

Recent calcium balance studies in adult patients with chronic kidney disease show that neutral calcium balance is achieved with calcium intake near the recommended daily allowance. Increases in calcium through diet or supplements cause high positive calcium balance, which may put patients at risk for vascular calcification. However, heterogeneity in calcium balance exists among these patients.

**Summary:**

Given the available calcium balance data in this population, it appears clinically prudent to aim for recommended calcium intakes around 1000 mg/day to achieve neutral calcium balance and avoid adverse effects of either negative or positive calcium balance. Assessment of patients’ dietary calcium intake could further equip clinicians to make individualized recommendations for meeting recommended intakes.

## Introduction

Patients with chronic kidney disease (CKD) have marked disruption in bone and mineral metabolism resulting in a complex disorder that has been termed CKD-mineral bone disorder (CKD-MBD). Perturbations begin in the earliest stages of the CKD and worsen with progressive kidney disease [1]. The biochemical alterations of CKD-MBD include elevated fibroblast growth factor-23 (FGF23) and parathyroid hormone (PTH), decreased 1,25-dihydroxyvitamin D (1,25D), elevated serum phosphate, and decreased serum calcium. Additionally, decreased calcium absorption and decreased urinary calcium excretion are observed, as well as heterogeneous bone disease and excessive vascular and soft tissue calcification. CKD-MBD is associated with an increased fracture risk and higher rate of cardiovascular events and cardiovascular-related deaths [2]. However, the underlying disease process is not completely understood, the initiators of the observed abnormalities are unclear, and definitive therapies are lacking. Both negative and positive calcium balance pose potential health threats in CKD-MBD: negative balance may increase risk for osteoporosis and fracture, and positive balance may increase risk for extraskeletal calcification and cardiovascular events. However, it is unlikely that negative or positive calcium balance alone is the initiating factor and it is unproven, although clinically plausible, that negative or positive calcium balance contributes to CKD-MBD disease progression in adults. The purpose of this review is to examine the available literature on calcium balance in CKD, discuss knowledge gaps and the future research needs in this area, and propose practical recommendations based on current available evidence.

## Assessing Calcium Balance

The definition of calcium balance is relatively simple: it is whole-body calcium retention or deficit calculated by subtracting total body calcium losses from total calcium inputs. However, the physiological processes controlling calcium balance make it more complex, particularly in CKD [3]. Further, conducting calcium balance studies is laborious and there are numerous important considerations and challenges in designing and executing these studies. There are also specific challenges unique to the CKD population. The first tenet of calcium balance studies is that research subjects must be in steady state, which implies no major fluctuations in inputs or outputs during the duration of the balance period. This necessitates the use of a tightly controlled and stable dietary intake that starts at least 1 week prior to taking balance measurements [4]. By using the non-absorbable fecal marker polyethylene glycol (PEG), the fecal calcium:PEG excretion ratio can be calculated and used to determine when subjects have equilibrated to a new controlled dietary calcium intake level (i.e., when the fecal calcum:PEG ratio stabilizes). This has been shown to occur after 6 days in adults, which provides the basis for the recommended minimum 1 week run-in on a controlled diet before beginning balance measurements [4]. The need for steady state precludes performing formal balance studies in patients on dialysis, since dialysis continually disrupts calcium balance and may promote fluxes in soft tissue and bone mineral content so that true steady state is never achieved. The second important tenet of calcium balance studies is that there must be very controlled dietary intake and very accurate measurements of all inputs and outputs.

In calcium balance studies, diets should be controlled to provide consistent amounts of calcium, but also other nutrients known to affect calcium balance such as phosphorus, sodium, and magnesium. Nutrient databases provide the starting point for designing any controlled diet, but it is important to note they are fraught with error, and pre-study composite meals must be chemically analyzed for nutrient content, and adjustments made to reach target values. Sophisticated design of diets for balance studies typically requires the expertise of registered dietitians who specialize in research diet design. Ideally, duplicate meals are also prepared alongside the meals given to subjects throughout the balance studies, and these duplicate meals are analyzed to provide the most accurate measurement possible of actual dietary intake. Additionally, subjects must be expected to eat only and all of study meals during balance studies, and when compliance is not 100%, uneaten food should ideally be weighed back, chemically analyzed, and accounted for in balance calculations. Calcium intake from supplements and medications must also be stable, controlled, and accounted for.

Complete collections of bodily excretions are required for accurate balance assessment. This includes all urine and feces, and in the most stringent studies, may also include losses from sweat, menstruation, ejaculate, saliva, and tears. When only urine and feces are collected, often the more insensible losses are either assumed to be minimal, similar between comparison groups, not affected by interventions, or estimated from other research papers. Reasonable stool regularity is another requirement for accurate balance calculations, and stool frequency also dictates that balance periods be sufficiently long to ensure that fluctuations in fecal output are able to be averaged over the balance study. Fecal markers such as PEG can be used to determine completeness of fecal collections [4, 5] although the technique is also error-prone when used to adjust fecal calcium output. Calcium balance studies of at least 1 or 2 weeks (post-diet run-in) are ideal [4]; however, there have been some successful studies of shorter duration [6•]. With this level of control, it is obvious why balance studies must be conducted in an inpatient environment of a clinical research center.

Isotopic calcium tracers are useful in augmenting balance studies and provide calcium kinetic data that includes estimated rates of calcium transport between body pools. Use of the gamma-emitter ^47^Ca is particularly useful as it allows for calcium balance studies that include whole-body counting. Importantly, neither serum ionized nor total calcium is reflective of whole-body calcium balance and cannot be used to estimate calcium balance in healthy subjects or patients with CKD [6•, 7•, 8].

## Implications of Negative, Neutral, and Positive Calcium Balance

Approximately 99% of the body’s calcium is stored in bones and teeth as hydroxyapatite (Ca_10_(PO_4_)_6_(OH)_2_) [9], the calcium-phosphate crystal lattice that makes up the majority of bone mineral content and contributes to bone strength. Thus, calcium balance is often used as a proxy of bone balance, and appropriate calcium balance values must be viewed in context of what is expected to be happening at the level of the skeleton. For example, in healthy adults, calcium balance and bone balance are generally assumed to be neutral. On the other hand, growing children are appropriately in high positive calcium balance (the positive calcium influx being incorporated into newly formed bone), corresponding to their rapid rate of skeletal accretion [10, 11]. Older adults and particularly post-menopausal women might be expected to be in negative calcium balance, reflecting negative bone balance and bone loss [12], as post-menopausal osteoporosis is foremost a disease of loss of bone mass rather than net negative calcium balance from low calcium intake. Simply from a bone perspective, one could conclude that negative calcium balance = bad (i.e., bone loss) and positive calcium balance = good (i.e., bone gain). However, for people who are not in skeletal anabolism, positive calcium balance may instead be indicating soft tissue deposition. For patients with CKD, both negative and positive calcium balance carry concerns. Negative calcium balance favors loss of bone mineral, the risk for a specific mineralization defect, increased risk for bone fragility fractures, and consequent morbidity and mortality, whereas positive calcium balance favors soft tissue calcification, consequent cardiovascular events, and related morbidity and mortality. Thus, in the adult CKD patient, neutral calcium balance appears to be the most desirable status to minimize risk of either adverse bone or vascular consequences. The subsequent sections will review calcium metabolism, available data from calcium balance studies, current calcium recommendations and intakes, and strategies for achieving a neutral calcium balance in adult CKD patients.

## Calcium Metabolism

In normal physiology, calcium metabolism is regulated through hormonal control of a three-tissue axis of intestine, kidney, and bone to tightly control serum ionized calcium within a narrow range. The two primary hormones involved are 1,25D (“active vitamin D”/calcitriol) and PTH. Low serum ionized calcium is sensed by the calcium-sensing receptors on the parathyroid gland, which stimulates PTH synthesis and secretion. PTH exerts several effects to raise serum ionized calcium: PTH (1) acts on the kidney proximal tubules to increase renal reabsorption of calcium, (2) stimulates bone osteoclast activity to increase bone resorption and calcium release, and (3) increases the renal enzyme, 1-α-hydroxylase (CYP27B1), responsible for the conversion of 25-hydroxyvitamin D (25D) to the most active form of 1,25D. The main role of 1,25D in correcting low serum ionized calcium is to increase intestinal calcium absorption.

Intestinal calcium absorption occurs by (1) a saturable, transcellular, active transport/facilitated diffusion component which is largely regulated by 1,25D acting through the transcriptional actions of the vitamin D receptor (VDR) and (2) an unsaturable, paracellular, passive diffusion component that is generally considered unregulated and linearly related to dietary calcium load. More recently, there is some evidence of potential regulation of the passive component of calcium absorption as well [13]. The relative importance and contributions of these two components of calcium absorption vary by intestinal segment depending on VDR levels and residence time. Active calcium absorption is highest in the proximal small intestine, followed by the large intestine, which parallels the abundance of VDR expression. It is estimated, primarily from rodent studies, that the large intestine accounts for a small portion (<10%) of total calcium absorption [14]. Evidence from human studies of patients with small bowel resection with or without a partially preserved colon also show the capability of the colon to help preserve calcium absorption [15, 16].

Calcium intake is an important factor affecting calcium absorption. When calcium intake is low, elevations in PTH and 1,25D act to increase the active absorption of calcium so that the fractional absorption of calcium is increased. However, absolute calcium absorption can still be low due to the saturable nature of active transport, even when vitamin D levels are sufficient. When calcium intake is insufficient, the bone calcium reserve is sacrificed in an effort to maintain serum calcium within (or toward) the normal range. Alternatively, high calcium intake suppresses PTH and the conversion of 25D to 1,25D, thus lowering active calcium absorption. At high calcium intakes, absolute calcium absorption is high, while fractional absorption is lower than with low calcium intakes. High calcium intake can also overcome the effects of vitamin D deficiency on bone, as evidenced by high calcium rescue diets for VDR knockout mice which completely rescue the bone phenotype in these animals [17]. This is achieved by bypassing the relative importance of the vitamin D-regulated active component of absorption and increasing calcium absorption through increased paracellular passive transport. In CKD, serum 1,25D levels are decreased due to the effects of FGF23 decreasing the conversion of 25D to 1,25D by inhibiting the 1-α-hydroxylase enzyme [18]. Thus, patients with CKD are at risk for low calcium absorption due to decreased levels of 1,25D and low active calcium transport. However, because active vitamin D-regulated calcium absorption is saturated at relatively low calcium intakes [19], even with vitamin D deficiency, high calcium intakes can result in normal to high absolute calcium absorption. In addition, there is evidence that intestinal epithelial cell tight junction proteins are altered in CKD, potentially affecting paracellular transport [20].

It has been previously suggested that calcium absorption may be severely impaired in early CKD based on very low observed urinary calcium excretion in these patients [21•]. However, evidence from calcium balance and kinetic studies illustrate that, despite very low urine calcium levels, patients with moderate (stage 3/4) CKD have fractional calcium absorption rates similar to healthy adults when dietary calcium is adequate [7•]. This supports studies dating back decades that have shown through radioisotopic tracer studies that calcium absorption is positively related to renal function, where it remains normal in earlier stages of the disease and is impaired as patients progress into the later stages of CKD [22–25].

## Calcium Balance Studies in CKD

**Fig. 1:**
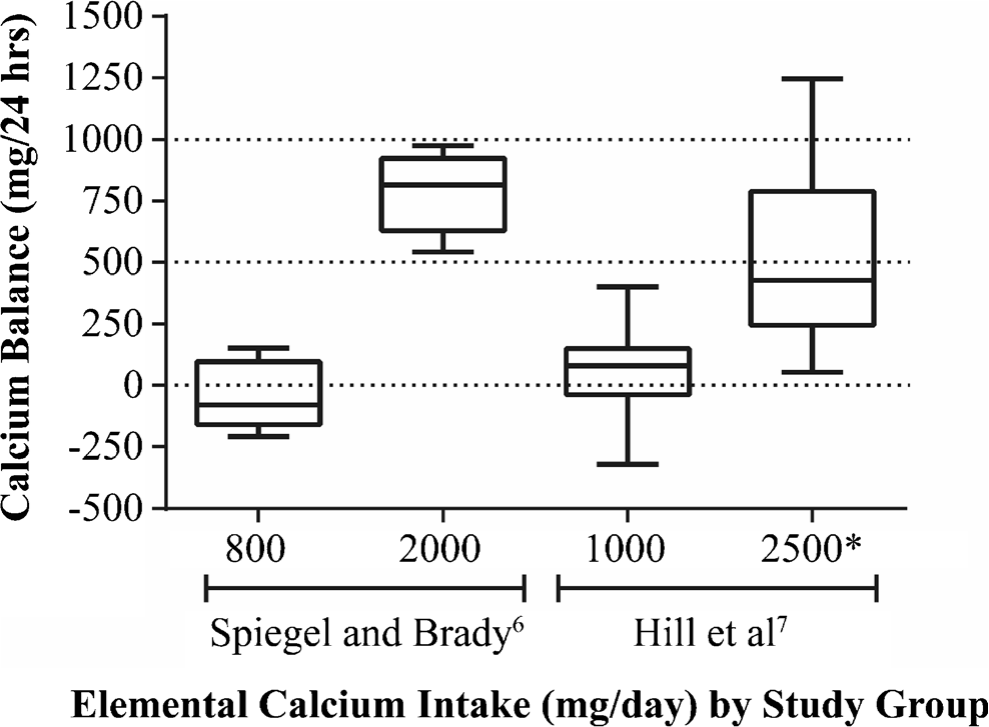
Calcium balance by elemental calcium intake in adult patients with CKD [6•, 7•]. The *bottom* and *top* of each box represent the 25th and 75th percentile, respectively; the *horizontal line* in each box represents the median, and the *bars* represent the range. *1500 mg was from calcium carbonate

Due to the need for steady state to conduct balance studies, patients with end stage renal disease (ESRD) undergoing dialysis are deemed not appropriate for formal balance studies. Two recent studies investigated the influence of calcium intake on calcium balance in patients with moderate (stage 3/4) CKD [6•, 7•]. Spiegel and Brady [6•] studied calcium balance in six patients with CKD and six healthy control individuals on two levels of dietary calcium intake, 800 and 2000 mg/day elemental calcium. Subjects participated in a randomized cross-over study that included two phases consisting of a 9-day run-in period when subjects consumed their assigned controlled diet, followed by a 48-h balance study conducted in an inpatient clinical research center setting. In another study, Hill et al. [7•] conducted calcium balance studies in eight patients with CKD consuming a controlled diet of 1000 mg/day elemental calcium with or without an additional 1500 mg/day elemental calcium from calcium carbonate given with meals. Subjects consumed their controlled diet plus calcium or placebo for 1 week as outpatients, and then they were admitted to a clinical research center as inpatients for 2-week balance studies in a randomized cross-over design. Both studies showed that average calcium balance in patients consuming 800–1000 mg/day elemental calcium was near neutral, and that increasing calcium intake by diet or by calcium carbonate pills produced a large positive calcium balance. An important detail is that on the lower calcium intakes in both studies, patients with moderate CKD were heterogeneous in their overall calcium balance, with some in negative, (virtually) neutral, or slightly positive balance (Fig. 1). In contrast, all CKD patients on the higher calcium intakes were in positive balance (Fig. 1). In both studies, patients with CKD had very low urine calcium output that remained low and nearly unchanged even though calcium intake was more than doubled. As noted above, calcium absorption from isotopic kinetic studies [7•] showed that fractional calcium absorption in the CKD patients was similar to healthy adults, indicating that the low urine calcium observed was not the result of low calcium absorption. Both studies also again demonstrated that serum calcium is not reflective of whole-body calcium balance, as high calcium intake increased whole-body calcium balance while serum calcium remained unchanged.

## Calcium Intake in Adult CKD Patients

The Institute of Medicine set new Dietary Reference Intakes (DRI) for the general population for calcium in 2010 [26]. The Tolerable Upper Limit (UL) set for calcium was 2000 mg/day. This is the same level that is suggested by the KDOQI guidelines (guidelines 5.5 & 6.4) as a maximum total amount of calcium from dietary sources plus calcium-based binders for patients with stage 3–5D CKD [27]. The KDIGO guidelines do not suggest limits for dietary calcium intake or any maximum level of total intake. However, for stage 3–5D CKD, KDIGO guidelines “recommend restricting the dose of calcium-based phosphate binders … in the presence of persistent or recurrent hypercalcemia” and “suggest restricting the dose of calcium-based phosphate binders in the presence of arterial calcification and/or adynamic bone disease and/or if serum PTH levels are persistently low” (guideline 4.1.5) [28]. Updated 2017 KDIGO guidelines for CKD-MBD (http://kdigo.org/home/guidelines/ckdmbdupdate) suggest limiting calcium-based phosphate binders for all patients with CKD stages 3a–5D.

The aforementioned balance studies [6•, 7•], though small in sample size, are consistent in showing that 2000 mg/day calcium (the UL for the general population, and the maximum level set by KDOQI) is too high for even moderate-stage CKD. Even though these studies did not include patients with ESRD, it can be reasonably assumed that they would have at least as great a risk for positive calcium balance with high calcium intake due to further impaired or absent urine output. This assumption is supported by modeling data in ESRD patients suggesting that these patients have a positive change in extracellular fluid calcium when elemental calcium intake exceeds 1500 mg/day, indicating risk for calcium retention [29]. Thus, the present data suggests that a more moderate target for dietary calcium intake should be recommended in CKD, around 800–1000 mg/day, which is close to the Recommended Daily Allowance (RDA) DRI of 1000–1200 mg/day (varying by age) for healthy adults [26].

The choice of whether or not to prescribe calcium supplements, calcium-based phosphate binders, or other medications containing calcium or to increase calcium from food in patients with CKD should depend on the baseline calcium intake of the individual patient. A recent cross-sectional study [30] that evaluated 3-day diet records in hemodialysis patients (*n* = 54) and non-CKD elderly adults (*n* = 47) found that calcium intakes were low (∼500 mg/day) compared with the DRI, but not different between the patients and the controls when all subjects were included. But, when only “adequate reporters” (a small subset of only *n* = 11 patients and *n* = 17 controls) were included, the average intake rose to ∼800 mg/day, but was also not different between groups. This is similar to a previous study in CKD patients (*n* = 117) who participated in the Lipid Lowering and Onset of Renal Disease (LORD) trial [31]. Four-day diet records were analyzed to determine nutrient intake and compared with dietary intake guidelines. The mean daily intake of calcium was ∼700 mg/day in all participants, but rose to 940 mg/day when only “valid reporters” (*n* = 33) were included. These mean intakes suggest that some patients are achieving adequate, but not excessive intake of calcium and in fact are similar to the general population [32]. However, in both of these studies, the standard deviations around the means were >300 mg/day. This points toward significant variability in calcium intakes among CKD and dialysis patients. These studies also speak to the importance of the influence of underreporting in estimating dietary calcium intake from diet records, which decreased the mean intake by ∼200 mg/day in both studies.

For patients with adequate calcium intakes of 800–1000 mg/day, clinicians might avoid recommending additional calcium supplements or prescribing calcium-containing medications. Patients with greater total calcium intakes (>approx. 1000 mg/day) may be advised to decrease calcium intake. On the other hand, patients with lower calcium intakes might be recommended to increase calcium through foods, supplements, or calcium-containing medications with the goal of achieving an estimated neutral calcium balance. Calcium-rich foods include dairy, dark green leafy vegetables, calcium-set tofu, and calcium-fortified orange juice. Bioavailability of calcium from these sources varies and is expressed in cup-equivalents to milk in Table 1 [33]. Other nutrients in these foods should also be taken into consideration, especially potassium and phosphorus, which are often restricted in patients with CKD. Dairy is particularly challenging for including in many patients’ diets, as it is high in both potassium and phosphorus, but modest amounts can usually be incorporated while keeping within dietary restrictions. Calcium supplements are a second option for increasing calcium intake. Given with meals, calcium supplements also can serve as dietary phosphate binders. The most common calcium-based phosphate binders in the USA are calcium acetate, calcium carbonate, or magnesium carbonate/calcium carbonate. Calcium citrate is a popular calcium supplement due to its relatively high bioavailability, but it is not recommended in CKD patients due to potential for citrate to increase aluminum absorption [27]. The elemental calcium content of calcium supplements/calcium-based binders varies as shown in Table 2 [37]. Other calcium-containing medications might also serve as an alternative way to improve calcium balance in patients with estimated low calcium intake and potentially negative balance, as a secondary benefit to their primary use. This is largely limited to calcium carbonate in antacid medications. Other drugs, such as atorvastin, contain calcium in minute amounts so small that it would not alter overall calcium balance. An exception is the relatively new potassium-binder patiromer, which contains calcium and is used in patients with CKD for treatment of hyperkalemia. Calcium is the cation used to exchange for potassium in this polymer. While the polymer itself is non-absorbable, some of the released calcium may be available for absorption. Recent data [38] has shown that patiromer (at a dose of 25.2 g/day) increased urinary calcium excretion in healthy people by 73 mg/day—implying a modest increase in gastrointestinal calcium absorption with the drug. In addition, urinary phosphate decreased with patiromer in healthy adults, suggesting some phosphate binding presumably by the released calcium in addition to its primary role as a potassium binding. It is important to note that patiromer is approved for treatment of hyperkalemia, and that the use of patiromer as a calcium source or phosphate binder is not part of the indication or prescribing information.

**Table 1:** Comparing sources for absorbable calcium

Source	Serving size^a^ (g)	Calcium content^b^ (mg/serving)	Estimated absorption efficiency^c^ (%)	Absorbable Ca/serving^d^ (mg)	Servings needed to = 1 cup milk
Foods					
Milk	240	290	32.1		1.0
Beans, pinto	86	44.7	26.7	11.9	8.1
Beans, red	172	40.5	24.4	9.9	9.7
Beans, white	110	113	21.8	24.7	3.9
Bok choy	85	79	53.8	42.5	2.3
Broccoli	71	35	61.3	21.5	4.5
Cheddar cheese	42	303	32.1	97.2	1.0
Cheese food	42	241	32.1	77.4	1.2
Chinese cabbage flower leaves	85	239	39.6	94.7	1.0
Chinese mustard green	85	212	40.2	85.3	1.1
Chinese spinach	85	347	8.36	29	3.3
Kale	85	61	49.3	30.1	3.2
Spinach	85	115	5.1	5.9	16.3
Sugar cookies	15	3	91.9	2.76	34.9
Sweet potatoes	164	44	22.2	9.8	9.8
Rhubarb	120	174	8.54	10.1	9.5
Whole wheat bread	28	20	82.0	16.6	5.8
Wheat bran cereal	28	20	38.0	7.54	12.8
Yogurt	240	300	32.1	96.3	1.0
Fortified foods					
Tofu, calcium-set	126	258	31.0	80.0	1.2
Orange juice with Ca citrate malate	240	300	36.3	109	0.88
Soy milk with tricalcium phosphate	240	300	24	72	1.3
Bread with calcium sulfate	16.8	300	43.0	129	0.74

Reprinted with permission from Springer Publishing [33]

^a^Based on a one-half cup serving size (∼85 g for green leafy vegetables) except for milk and fruit punch (1 cup or 240 mL) and cheese (1.5 oz)

^b^Taken from Refs. [34] and [35] (averaged for beans and broccoli processed in different ways) except for the Chinese vegetables which were analyzed in our laboratory

^c^Adjusted for load using the equation for milk (fractional absorption = 0.889–0.0964 ln load) then adjusting for the ratio of calcium absorption of the test food relative to milk tested at the same load, the absorptive index [36]

^d^Calculated as calcium content × fractional absorption

**Table 2:** Elemental calcium content of calcium supplements [37]

	Elemental calcium
Calcium carbonate	40.0%
Calcium chloride	27.2%
Calcium acetate	25.3%
Calcium citrate	21.1%
Calcium lactate	13.0%

## Conclusions

A great deal of confusion and controversy exist regarding calcium in CKD and dialysis. It is clear that with advancing kidney disease, the kidneys are no longer able to increase urine calcium excretion, and this removes an important safety mechanism to prevent calcium excess in patients with CKD. However, it is also well-known that 1,25D levels fall with advancing CKD and intestinal calcium absorption becomes increasingly dependent on a positive gradient to maintain even neutral flux, as calcium can be both absorbed and lost via the gastrointestinal tract. Until more is understood about the disease process, it seems clinically reasonable to try to maintain neutral calcium balance throughout the stages of CKD, as calcium deficiency could lead to excessive bone loss and secondary hyperparathyroidism [39] and calcium excess could accelerate vascular and soft tissue calcification [40, 41]. Balance studies suggest that patients are, on average, in neutral calcium balance while consuming 800–1000 mg/day. This is therefore a sensible starting point for dietary calcium intake recommendations. There is no evidence that consuming less dietary calcium provides benefit, and it could cause harm by worsening bone health and driving secondary hyperparathyroidism. If calcium intake is low in CKD or dialysis patients, counseling seems reasonable in an attempt to achieve 800–1000 mg/day intake. Challenges come in accurately estimating patients’ usual dietary calcium intakes and in identifying those patients who may require higher or lower intakes than the 800–1000 mg/day calcium range, or those patients for whom a positive or negative balance may be desirable. Dietary assessment by dietitians through multiple 24-h recalls, diet records, or calcium-specific food frequency questionnaires is a first step to better understand individual patient’s typical intakes. The validated NIH short calcium food frequency questionnaire [42] or the Dialysis-FFQ [43•] may provide a quick solution in this regard for busy clinicians. For patients who fall below 800–1000 mg/day, modest increases in calcium from calcium-rich food sources, calcium supplements, calcium-based phosphate binders, or other calcium-containing medications might be considered. However, further research is needed before claims of any benefit of these approaches to clinical outcomes can be made. Other future research needs include identifying predictors of calcium balance in patients with CKD or alterative tools to performing classical mineral balance studies so that whole-body calcium balance might be estimated in clinical settings. In addition, there is a need to define the optimal calcium intake for the growing child with CKD. Clearly, serum calcium and urine calcium alone, or changes in these ions, cannot serve as proxy measures of whole-body calcium balance.
